# Effectiveness of a multi-level intervention to reduce men’s perpetration of intimate partner violence: a cluster randomised controlled trial

**DOI:** 10.1186/s13063-020-4185-7

**Published:** 2020-04-25

**Authors:** Nicola J. Christofides, Abigail M. Hatcher, Dumisani Rebombo, Ruari-Santiago McBride, Shehnaz Munshi, Angelica Pino, Nada Abdelatif, Dean Peacock, Jonathan Levin, Rachel K. Jewkes

**Affiliations:** 1 Faculty of Health Sciences, School of Public Health, University of the Witwatersrand, 27 St Andrews Rd, Parktown, Johannesburg, 2193 South Africa; 2 Division of HIV, Infectious Disease, and Global Medicine, University of California, SanFrancisco, USA; 3 Sonke Gender Justice, Juta Street, Braamfontein, Johannesburg, South Africa; 4 South African Medical Research Council, 1 Soutpansberg Road, Pretoria, South Africa

**Keywords:** Cluster randomised controlled trial, Community mobilisation intervention, Intimate partner violence

## Abstract

**Background:**

Men’s perpetration of intimate partner violence (IPV) limits gains in health and wellbeing for populations globally. Largely informal, rapidly expanding peri-urban settlements, with limited basic services such as electricity, have high prevalence rates of IPV. Evidence on how to reduce men’s perpetration, change social norms and patriarchal attitudes within these settings is limited. Our cluster randomised controlled trial aimed to determine the effectiveness of the Sonke CHANGE intervention in reducing use of sexual and/or physical IPV and severity of perpetration by men aged 18–40 years over 2 years.

**Methodology:**

The theory-based intervention delivered activities to bolster community action, including door-to-door discussions, workshops, drawing on the CHANGE curriculum, and deploying community action teams over 18 months. In 2016 and 2018, we collected data from a cohort of men, recruited from 18 clusters; nine were randomised to receive the intervention, while the nine control clusters received no intervention. A self-administered questionnaire, using audio-computer assisted software, asked about sociodemographics, gender attitudes, mental health, and the use and severity of IPV. We conducted an intention-to-treat analysis at the cluster level comparing the expected risk to observed risk of using IPV while controlling for baseline characteristics. A secondary analysis used latent classes (LCA) of men to see whether there were differential effects of the intervention for subgroups of men.

**Results:**

Of 2406 men recruited, 1458 (63%) were followed to 2 years. Overall, we saw a reduction in men’s reports of physical, sexual and severe IPV from baseline to endpoint (40.2% to 25.4%, 31.8% to 15.8%, and 33.4% to 18.2%, respectively). Intention-to-treat analysis showed no measurable differences between intervention and control clusters for primary IPV outcomes. Difference in the cluster-level proportion of physical IPV perpetration was 0.002 (95% confidence interval [CI] − 0.07 to 0.08). Similarly, differences between arms for sexual IPV was 0.01 (95% CI − 0.04 to 0.06), while severe IPV followed a similar pattern (Diff = 0.01; 95% CI − 0.05 to 0.07). A secondary analysis using LCA suggests that among the men living in intervention communities, there was a greater reduction in IPV among less violent and more law abiding men than among more highly violent men, although the differences did not reach statistical significance.

**Conclusion:**

The intervention, when implemented in a peri-urban settlement, had limited effect in reducing IPV perpetrated by male residents. Further analysis showed it was unable to transform entrenched gender attitudes and use of IPV by those men who use the most violence, but the intervention showed promise for men who use violence less.

**Trial registration:**

ClinicalTrials.gov, NCT02823288. Registered on 30 June 2016.

## Introduction

Men’s perpetration of intimate partner violence (IPV) against female partners limits gains in health and wellbeing for women globally. It is estimated that around one-third of women experience IPV in their lifetime [1]. Within a community, inequitable gender attitudes, roles, values, entitlements and norms structurally frame and enable violence against women and girls. Masculinities are an integral part of this structure. Those rooted in patriarchy and an entitlement and expectation to dominate and control women, enacted through exploitative sexual behaviours and relationships, are most commonly associated with IPV [2, 3]. Peri-urban settlements in sub-Saharan Africa have much higher rates of IPV than seen elsewhere [4, 5]. In contexts of high unemployment, informal housing, food insecurity and other forms of poverty, epitomised by peri-urban, largely informal settlements, men find it very difficult to achieve successful manhood, as constructed by their wealthier peers emphasising providing for a home and having material possessions, and instead may enact models of masculinity characterised by dominance [6–8]. While some men persist in their difficult circumstances pursuing more benign patriarchal models, others are enticed by hypermasculine models of manhood that most strongly emphasise control over women, heterosexual prowess, coupled with access to resources through crime. The latter are most conspicuously violent towards women [9, 10]. Interventions to address these drivers of IPV perpetrations often address all participants in a similar way. Better understanding how different sub-groups of participants respond to an intervention would inform future tailoring.

In the last two decades, activists and researchers have partnered to develop and test growing numbers of interventions to prevent IPV, including those that seek to change social norms on gender and violence through community mobilisation [11–13]. New evidence from rigorous evaluations of community-mobilisation and outreach interventions show promise in reducing women’s experiences of IPV. Notable among these are SASA! Activist Kit for Preventing Violence against Women and HIV and Safe Homes and Respect for Everyone (SHARE) both carried out in Uganda [14, 15]. These community mobilisation and outreach interventions reduced women’s experiences of IPV, significantly in the case of SHARE [14, 15], but were not found to reduce men’s use of IPV against female partners.

Sonke Gender Justice (Sonke), a South African non-governmental organisation, has been running gender transformative, community-based programmes since 2006. The core Sonke intervention, ‘One Man Can’, is premised on mobilising communities to take action to bring about more gender equitable social norms and positive parenting [16, 17]. Activities include workshops and other reflective activities to challenge harmful gender norms and educate men about gender-based violence and HIV risks. The theory underpinning the intervention is that through community outreach and advocacy, harmful values and practices can be transformed toward gender equity and thereby reduce HIV risk and gender-based violence [18]. Sonke, with research partners, has tested the intervention in rural Mpumalanga, South Africa, in a study reported in parallel with ours which found that the intervention shifted men’s gender attitudes and women’s experience of IPV [19]. However, since Sonke was implementing the intervention in urban settings in and around Johannesburg, there also was a need to evaluate whether the intervention was effective in an urban setting. There was an opportunity to further enhance and test the intervention to address men’s use of IPV and non-partner sexual violence.

Our cluster randomised controlled trial aimed to determine the effectiveness of the Sonke Community Health Action for Norms and Gender Equity (CHANGE) intervention to prevent use of sexual and or physical violence against an intimate partner and reduce the severity of perpetration by men aged 18–40 years living in a peri-urban South African settlement over 2 years of follow-up. A sub-analysis sought to explore whether the intervention had an effect on different groups of men, based on baseline characteristics.

## Methods

### Study setting

The trial was conducted in a semi-formal ‘township’ located near Johannesburg, South Africa. The peri-urban settlement took form in the mid-1990s as a result of rapid urbanisation, with many South Africans moving closer to cities to seek employment. Most residents live in government-subsidised housing (about one-third) and informal tin shacks. Few exact population estimates exist; the census of 2011 estimated the population to be 140,000, but most assume the ‘township’ has 250,000–500,000 residents, including high numbers of migrants from Southern African Development Community (SADC) countries. While electrification and water are available for large parts of the area, there are parts where residents lack access to basic services such as running water, sewerage and rubbish removal. The study was approved by the University of the Witwatersrand Human Research Ethics Committee (M150443). Our protocol is registered at clinicaltrials.gov (NCT02823288) and a full account of the methods is provided elsewhere [20].

### Intervention activities

**Fig. 1:**
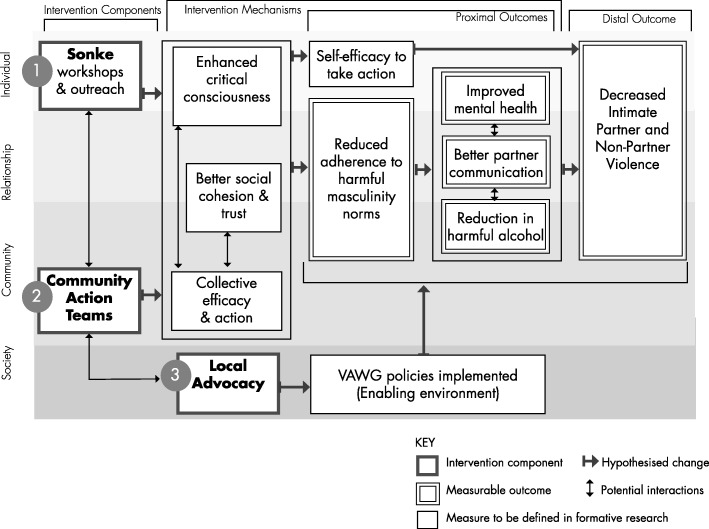
Intervention Theory of Change

The Sonke CHANGE Intervention was informed by a theory of change. Figure 1 shows the model which outlines how the intervention components were intended to combine to lead to personal change, community shifts in social norms and the creation of an enabling environment. It was implemented over a period of 18 months (April 2016 to November 2017) by the non-governmental organisation Sonke, with a multi-level approach. The activities were carried out by a full-time manager and three male and three female community mobilisers recruited from the community who received extensive training over several months before the launch of the intervention and ongoing professional development throughout its duration.

In addition, male and female volunteers who formed Community Action Teams (CATs) received training through 1-week workshops. The training aimed to deepen the content of the workshops on gender equity. CATs committed to conducting activities for 5 h per week. These mostly entailed having discussions with peers (see door-to-door conversations and mini-workshops). CAT members volunteered over the 18 months that the intervention took place, during which periodic training workshops were held. Their numbers fluctuated and a total of 61 were recruited over the intervention period.

The Sonke CHANGE intervention as implemented in the peri-urban setting was designed to focus on three dimensions: (1) community mobilisation through the creation of CATs; (2) peer outreach and education which focused on providing information on human rights, equitable gender attitudes, alcohol abuse and gender-based violence (including IPV); and (3) local advocacy for change. The in-depth community mobilisation and education consisted of a series of six 2-day (12 h) workshops conducted by community mobilisers. The themes included: gender; gender socialisation; gender roles; gender power and violence; gender and sexuality; gender and HIV; healthy relationships; and alcohol abuse; with gender-based violence discussed as a cross-cutting issue. The exercises were developed and documented in a manual, spanning 72 h of training (six 2-day workshops), but each workshop theme was delivered as a stand-alone activity and was not intended to be delivered as a ‘course’ for participants. Workshop participants were then encouraged to join CATs. With support and coordination by paid mobilisers, CATs were intended to be the vehicle for peer education and outreach activities, involving both men and women. Other activities comprised: door-to-door activities which involved community mobilisers and CATs engaging individuals or small groups where they congregated on the streets or in their homes; mini-workshops which took place over 2–3 h; murals which were used to engage men and women passing by in discussions similar to those that occurred during door-to-door activities; facilitated discussions in venues where men gather including taverns/bars; use of picture charts with a thematic discussion to follow; and community dialogues, which were conducted by both community mobilisers and CATs. All members of the communities in the intervention clusters were targeted through the activities, not only study participants. The reason for this was that the intervention was premised on mobilising the community, in other words, all community members should be reached either directly through participation in the activities described or indirectly through contact with community members reached through the intervention activities.

The implementation of the Sonke CHANGE intervention was monitored by project staff. Registers of participants were completed at every workshop. In addition, every other type of activity that was conducted by either a community mobiliser or CAT was captured on data forms which were later captured in an electronic database using Open Data Kit software.

The planned advocacy component aimed to support local government to implement community violence prevention measures, like reducing alcohol outlet density, improving community lighting and increasing community policing, and holding government to account for their obligations to ensure the criminal justice system met the needs of survivors and held perpetrators accountable. However, this advocacy component was less well articulated, and perhaps less well understood, and consequently only implemented to a very limited extent. Further details about the intervention are described elsewhere [20].

### Trial design

We conducted a two-arm cluster randomised controlled trial to measure the impact of the intervention on the primary and secondary study outcomes. The process of recruitment and randomisation is described in the published protocol [20]. Eighteen clusters, identified for the purposes of the trial, were randomised so that nine received the Sonke CHANGE community mobilisation and education intervention while nine were control neighbourhoods. Due to the informality of geographic boundaries within the peri-urban settlement, a cluster was defined as a neighbourhood bordered by or extending to a community landmark such as a church, community hall or communal water source. Clusters identified for inclusion in the study were not contiguous and each was bordered by a natural boundary (such as a stream) or by a distance of at least 400 m which served as a buffer in order to limit contamination between intervention and control neighbourhoods. Randomisation of clusters into the intervention or control arm was undertaken after the baseline data collection was completed. Randomisation was performed at a public event, where local leaders chose one cluster name from a bag until nine clusters were allocated to the intervention arm.

**Fig. 2:**
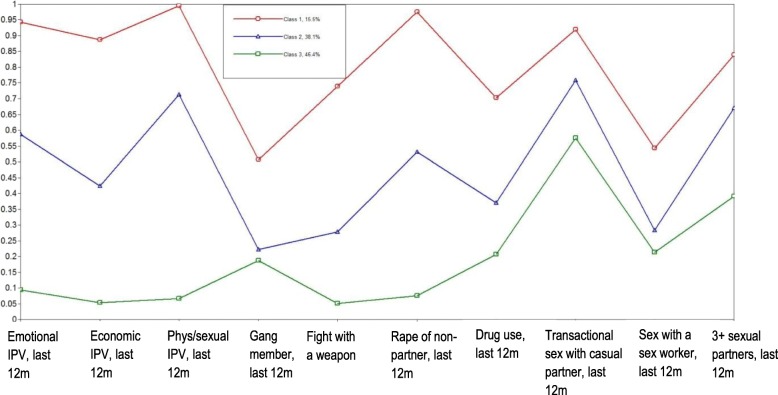
Three classes of men identified using latent class analysis at baseline

**Fig. 3:**
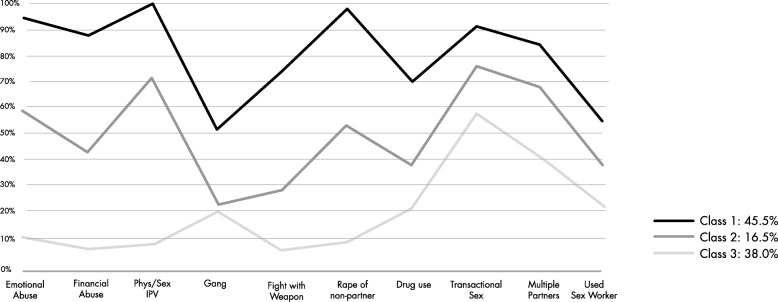
Effectiveness of the intervention for the three classes of men on the outcomes: perpetration of physical, sexual and severe interpersonal violence

Approximately 120–150 men aged 18–40 years who lived in the area for at least 12 months were recruited from each neighbourhood and followed up for 24 months after their enrolment in the study (Figs. 2 and 3). Participants were recruited by trial staff who went door-to-door in a cluster and this was supplemented by those living in the area who heard about the study from their friends and neighbours. Men were invited to take part in a written informed consent process and thereafter asked to complete a locator form which included contact names and numbers of the participant and important people in their lives (e.g. family members, partners and close friends). Contact with participants between data collection to maintain the cohort included sending text messages and phone calls. At the follow-up data collection, participants were contacted and asked to return to a data collection point in their neighbourhood to complete the questionnaire. Efforts to trace men at the endpoint included making multiple calls to their cell phones, and contacting their next of kin, their friends and friends of friends. The field work team went to addresses where men reported living, walked in the cluster and asked people if they knew the participant. In addition, data were collected from men who had moved out of the study site to other provinces, cities and neighbourhoods within Johannesburg. We verified the identity of the men through matching three separate points of data that included: their name, telephone numbers of the participant and their family and/or friends. Data from the 12-month follow-up are not presented as there were issues with data quality. We present data from the 24-month follow-up.

A process evaluation employing ethnographic methods was carried out alongside the trial over the period of the implementation of the Sonke CHANGE intervention between 2016 and 2018 [21]. The methods included participant observation and in-depth interviews with managers, intervention staff, CATs and participants. Some participants were interviewed at multiple timepoints over the period of implementation. The aim of the process evaluation was to explore how the intervention was conceptualised and delivered.

### Baseline and post-intervention assessments

Data collection was facilitated by trained research assistants and conducted in the language of participant choice (English, isiZulu, Xitsonga or Sepedi). The questionnaire was administered using audio-computer assisted data collection (ACASI) since there were sensitive questions around violence perpetration [22]. The use of ACASI prevented ethical dilemmas for the research team as it reduced the possibility that men would disclose criminal activity to the interviewer. No interviewer or researcher could examine responses to questions until the data were de-identified through the electronic data system. Data were uploaded to an encrypted server, purpose-built for this study.

### Measures

The long-term goal of the intervention was to reduce men’s use of physical and sexual intimate partner violence against women. The primary outcome, men’s reported violence towards an intimate partner, was measured using an adapted version of the questionnaire from the South African Medical Research Council’s Study on Men’s Health and Relationships [23, 24]. The questionnaire included eight items around physical and sexual violence. Primary outcomes were dichotomous: any use of physical violence and any use of sexual violence against a partner in the past 12 months. Use of severe sexual and/or physical violence was assessed by summing the categorical assessment of frequency and dividing the variable, such that IPV would be regarded as severe if there was any level of affirmative response to more than one question on physical and/or sexual violence in the past 12 months, or any response of ‘few’ or ‘many’ to any item on physical or sexual violence in the past 12 months.

Secondary outcome measures included harmful use of alcohol, which was measured using the Alcohol Use Disorders Identification Test (AUDIT), a 10-item scale designed to measure alcohol consumption and identify risks for alcohol abuse and dependence (Cronbach alpha = 0.82) [25]. Problem drinking was assessed as a score of ≥ 8 on the AUDIT. Perpetration of non-partner rape was measured using an adapted version of the questionnaire from the South African Medical Research Council’s Study on Men’s Health and Relationships [23, 24].

Gender attitudes were measured using the Gender Equitable Men’s Scale [26] and the Gender Norms scale on whether a man perceives that his community holds those beliefs [27] (Cronbach alpha = 0.85). Male controlling behaviour was measured using an adaptation of the Sexual Relationship Power and Control Scale (Cronbach alpha = 0.86) [28].

Parenting was included as a secondary outcome as the precursor to the CHANGE intervention, ‘One Man Can’, had found positive benefits on men’s parenting beliefs and practices [17]. Parenting was measured by the Parent-Child Conflict Tactics Scale, a series of items about parental psychological abuse and physical discipline of children [29]. The Cronbach alpha was 0.72 indicating adequate internal consistency. Transactional sex was measured using five items about transactional sex among casual partners [27].

Social cohesion was assessed using a measure from the Stepping Stones questionnaire (Cronbach alpha = 0.80) [30]. Depression was measured using the 20-item Center for Epidemiological Studies Depression (CES-D), a brief, validated screener based on the nine diagnostic criteria for DSM-IV depressive disorders (Cronbach alpha = 0.89) [31]. A score of 16 or higher on the CES-D denoted symptoms consistent with probable depression.

### Covariates

Partnership characteristics included basic demographics about sexual partners and sexual behaviour from the Stepping Stones questionnaire [30]. Socioeconomic status was assessed using items from the United Nations Multi-country Study on Men and Violence in Asia and the Pacific around education, marital status, household size and monthly income. Food security was measured using the Household Hunger Scale, a three-item measure developed by the Food and Nutrition Technical Assistance (FANTA) project [32]. Exposure to the intervention before baseline and in both intervention and control communities was measured through a series of questions that ask about awareness of Sonke Gender Justice, participation in workshops and other activities.

### Sample size

Based on a previous household survey of prevalence of men’s perpetration of IPV, we estimated that 12% of men would use physical or sexual violence towards a partner in the past 12 months [27]. We estimated the sample size based on a reduction if IPV to 7%. We carried out sample size calculations based on Moulton and Hayes (2009) for nine clusters per arm with a coefficient of variation (k) of 30%. The power calculation to detect the difference was based on approximately 150 participants per cluster in 18 clusters.

### Statistical analysis

All men with a recorded primary outcome at baseline (e.g. IPV) were included in the intention to treat analysis, regardless of whether they moved out of the area or moved to another cluster within the area. Men were included in the intention to treat (ITT) analysis whether they reported being aware of participating in the intervention or not. Since the number of clusters was small (n = 18) all analyses were done at the cluster level based on the Hayes and Moulton (2009) approach to analysing Cluster Randomised Controlled Trials (CRT) [33]. We compared the proportion of men perpetrating IPV in the nine intervention clusters compared to the nine control clusters at endpoint while adjusting for baseline levels of IPV and sociodemographic characteristics: age; educational attainment; number of children; and relationship status [33]. The difference between the intervention and control at the endpoint was reported. The difference in the mean proportions at the endpoint was compared using a t-test and the *p* value is reported. We used inverse probability weighting to adjust for missingness. In our sensitivity analysis using inverse probability weighting at the cluster level, we found a 0.03-point difference (95% CI: − 0.13 to 0.19; *p* = 0.69).

To analyse subgroup differences between men, latent class analysis (LCA) was used, as it is postulated that underlying latent subgroups can be identified by the probability of men having engaged in a range of behaviours indicated by a set of variables. LCA is a finite mixture model for categorical variables and has been used to define classes in research in South Africa with a similar population of men and in research from Asia/Pacific [2]. The theoretical underpinning for the class definition is that these variables are indicators for different masculine positions, as justified in Jewkes and Morrell [2]. The six variables that were considered to define the latent subgroups, or latent classes of men, were IPV perpetration in the last 12 months (emotional, economic, physical and sexual), ever having been a gang member, having fought with a weapon/had an illegal gun in the past 12 months, rape of a non-partner in the past 12 months, drug use in past 12 months, transactional sex with a casual partner in the past 12 months, sex with a sex worker and having had three or more sexual partners in the past 12 months. These variables were measured at baseline. The optimal number of classes was identified using several measures of fit to determine the best model, as well as what is the most ‘interpretable’ number of classes. The optimal number of classes was identified using several measures of fit to determine the best model, as well as what is the most ‘interpretable’ number of classes. The overall measures of fit that were used were the log-likelihood (LL), likelihood ratio chi-squared statistic (L^2^), Akaike Information Criterion (AIC), Bayesian Information Criterion (BIC), adjusted BIC (aBIC) and entropy. A key assumption of LCA is that there is local independence in the variables conditional on the latent class, so to test whether this assumption has been violated, the bivariate residual Pearson chi-squared statistic was used. This statistic measures the extent to which the observed association between two variables is reproduced by a model. The latent classes that were identified were used to examine differential treatment effects within each class, by fitting logistic regression models at the individual level for each of the three classes of men. The analyses were adjusted for baseline level of the IPV exposure, number of children, age, educational attainment and relationship status, and accounted for the clustered nature of the data.

## Results

**Fig. 4:**
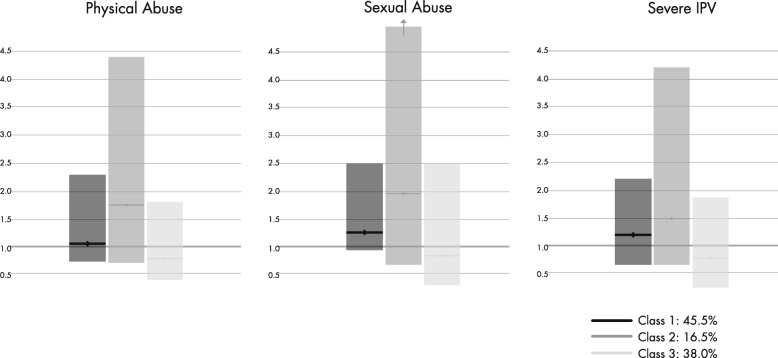
*Flow diagram* showing baseline recruitment, allocation (2016) and endpoint retention (2018)

We followed up 1508 men at 24 months (63%). Figure 4 shows that no clusters were lost over the study period and a similar proportion of men were followed up in the intervention and control clusters. The number of men followed up per cluster was in the range of 65–102. The proportion of men followed up in the intervention clusters was 63.1% and in the control clusters was 60.0%. Nineteen men had died over the period of the study (none due to the study) while 17 had been arrested. The main reason for men not being traceable at the endpoint was that they had moved away and were no longer reachable through any of the contacts that they had provided at baseline.

After 18 months, intervention activities had reached 14,878 women and men. Based on rapid household counts in each cluster, we estimated that the proportion of men in each of the nine intervention neighbourhoods reached at least once through an intervention activity was in the range of 30%–70%. The proportion reached by the more intensive 2-day workshops was lower, in the range of 6%–33% [21].

Table 1 shows the participants’ characteristics at baseline. The mean age of participants was 27.3 years in the intervention clusters and 27.8 years in the control clusters, the same proportion (39.2%) had completed their high school education successfully (received a matric), the majority of participants were partnered with 38.3% living with their partner in intervention clusters and 40.1% in control clusters, while 46.7% in intervention clusters and 42.0% in control clusters lived apart. The majority of participants were migrants, with 11.4% being cross-border migrants and 69.5% internal migrants from other provinces in the country in the intervention clusters, while in the control clusters 14.5% were cross-border migrants and 67.0% were from other provinces in the country. More than one-third (36.5%) of participants in the intervention and 37.5% in the control neighbourhoods were unemployed and a similar proportion (41.6% vs 41.3%) lived in informal housing and 55.9% in the intervention and 53.9% in the control communities reported food insecurity. At baseline, 40.8% of men in the intervention and 43.4% in the control neighbourhoods reported using physical IPV in their lifetime, while 43.5% in the intervention and 43.3% in the control clusters had used sexual IPV. There were no statistically significant differences between the intervention and control clusters in primary or secondary outcomes.

**Table 1:** Descriptive data on cohort at baseline by intervention and control arms

	Intervention	Control	
Characteristics and behaviours at baseline	Mean ± SD or n (%)	Mean ± SD or n (%)	*P* value
Age (years) (n = 2406)	27.3 ± 5.6	27.8 ± 5.7	0.04
High school education	451 (38.2)	457 (38.2%)	0.98
Relationship status			
-Partnered, living together	453 (38.3)	479 (40.1)	
-Partnered, living apart	553 (46.7)	502 (42.0)	0.04
-Single	178 (15.0)	213 (17.8)	
Cross-border migrant	136 (11.4)	174 (14.5)	0.03
Internal migrant	730 (69.5)	689 (67.0)	0.22
Lived in informal housing	494 (41.6)	498 (41.3)	0.86
Unemployed	423 (35.5)	450 (37.5)	0.29
Household hunger	663 (55.9)	647 (53.9)	0.32
Physical abuse	465 (39.5)	487 (40.0)	0.51
Sexual abuse	372 (31.7)	381 (32.0)	0.90
Severe IPV	376 (32.9)	393 (33.8)	0.64
Non-partner rape	409 (34.3)	402 (34.3)	0.99
Possible depression	543 (43.9)	500 (41.1)	0.17
Harsh parenting	321 (59.0)	315 (56.6)	0.41
Harmful alcohol use	594 (50.2)	585 (49.8)	0.87

*IPV* interpersonal violence, *SD* standard deviation

We compared the characteristics of those retained over the 24 months of follow-up and those lost to follow-up (Table 2). Participants who were lost to follow-up were less likely to have completed high school and were more likely to be single (not in a relationship), a cross-border migrant and live in informal housing. Although use of physical IPV at baseline was similar, men who were lost to follow-up were more likely to report use of sexual IPV (*p* = 0.02) during the past year, as well as using IPV against a partner multiple times (*p* = 0.004).

**Table 2:** Descriptive data on participants lost to follow-up over 24 months

	Retained over 24 months	Lost to follow-up	*P* value
Sociodemographics at baseline	Mean ± SD or n (%)	Mean ± SD or n (%)	
Age (years) (n = 2406)	27.4 ± 5.7	27.7 ± 5.6	0.20
High school education	557 (39.9)	331 (35.6%)	0.04
Relationship status			
Partnered, living together	559 (38.7)	373 (40.0)	
Partnered, living apart	685 (47.4)	370 (39.7)	< 0.001
Single	201 (13.9)	190 (20.4)	
Cross-border migrant	155 (10.7)	155 (16.5)	< 0.001
Internal migrant	730 (69.5)	689 (67.0)	
Lived in informal housing	577 (39.7)	415 (44.1)	0.04
Unemployed	540 (37.2)	333 (35.4)	0.36
Household hunger	795 (54.9)	515 (54.8)	0.94
Use of physical IPV in past year	558 (38.8)	394 (42.4)	0.08
Use of sexual IPV in past year	432 (30.0)	321 (34.7)	0.02
Severe IPV in past year	434 (31.1)	335 (36.9)	0.004

*IPV* interpersonal violence, *SD* standard deviation

We compared the cluster-level adjusted proportions of men using IPV in the past 12 months against a female partner in the intervention and control communities. Overall, we saw a reduction in men’s reports of past 12 months IPV perpetration from baseline (Table 1) to endpoint (Table 3) across all clusters. There were no statistically significant differences between the intervention and control communities on any of the three primary outcomes at the endpoint: perpetration of physical abuse against a partner; perpetration of sexual abuse against a partner; or perpetration of severe abuse against a partner (Table 3). Difference in the cluster-level proportion of physical IPV perpetration was 0.002 (95% CI: − 0.07 to 0.08). Similarly, differences between arms for sexual IPV was 0.01 (95% CI: − 0.04 to 0.06). While severe IPV followed a similar pattern (Diff = 0.01; 95% CI: − 0.05 to 0.07).

**Table 3:** Primary outcomes, differences in the cluster-level proportion of men using IPV against a female partner between the intervention and control arms at endpoint

	Endpoint n (%)		Endpoint – cluster-level proportions^a^ n (%)		Difference in proportion at endpoint (95% CI)		*P* value
	Intervention	Control	Intervention	Control			
Physical abuse	134 (17.54)	140 (18.97)	9 (27.68)	9 (27.89)	0.002	(−0.07 to 0.08)	0.95
Sexual abuse	115 (15.01)	122 (16.53)	9 (17.28)	9 (18.63)	0.01	(− 0.04 to 0.06)	0.58
Severe IPV	134 (17.54)	140 (18.97)	9 (20.46)	9 (21.41)	0.01	(−0.05 to 0.07)	0.74

^a^Cluster level proportions which aggregated individual-level data at endpoint adjusted for baseline levels of IPV and sociodemographic characteristics

*CI* confidence interval, *IPV* interpersonal violence

Table 4 shows the findings for the secondary outcomes. Again, there were no significant differences between the intervention and control communities on any of the secondary outcomes at the endpoint including: non-partner rape; use of transactional sex; depression symptoms; harmful use of alcohol; sexual power relations; gender attitudes; parenting; or social cohesion. We found that harmful use of alcohol as measured by the audit (with a cut point of 8) showed that men in the control communities reported lower levels compared to the intervention communities at the endpoint.

**Table 4:** Differences in the residuals of secondary outcomes between the intervention and control arms at endpoint controlling for baseline prevalence and sociodemographic characteristics

	Baseline		Endpoint		Differences in residuals at endpoint (95% CI)		*P* value
	Intervention	Control	Intervention	Control			
Non-partner rape (n (%))	402 (34.33)	409 (34.34)	190 (27.86)	201 (30.04)	−0.001	(−0.13 to 0.13)	0.98
Transactional sex (n (%))	529 (49.03)	515 (47.60)	298 (42.69)	282 (40.93)	−0.05	(− 0.19 to 0.09)	0.47
AUDIT (mean ± SD)	8.53 ± 6.43	8.62 ± 6.58	7.80 ± 6.40	7.73 ± 6.66	−0.06	(−0.16 to 0.04)	0.23
Problematic alcohol use (AUDIT 8) (n (%))	528 (57.83)	526 (57.36)	421 (55.39)	380 (52.13)	−0.09	(− 0.16 to 0.01)	0.02
CES-D (mean ± SD)	16.53 ± 8.26	17.15 ± 8.42	16.54 ± 8.34	16.93 ± 9.30	−0.04	(−0.11 to 0.02)	0.18
Possible depression CES-D 16 (n (%))	500 (41.12)	543 (43.86)	345 (28.37)	326 (26.33)	−0.11	(− 0.28 to 0.07)	0.22
GEMS (mean ± SD)	22.87 ± 6.60	23.16 ± 6.06	22.81 ± 6.23	22.05 ± 6.35	−0.05	(−0.12 to 0.03)	0.19
SRPS (mean ± SD)	19.70 ± 5.54	19.92 ± 5.16	19.38 ± 5.02	19.43 ± 5.32	−0.03	(− 0.13 to 0.07)	0.59
Parenting (parent–child conflict tactics scale) (n (%))	315 (56.55)	321 (59.01)	230 (56.79)	245 (58.47)	0.06	(−0.13 to 0.25)	0.53
Social cohesion (mean ± SD)	14.07 ± 3.11	14.43 ± 2.88	14.80 ± 3.16	14.85 ± 3.11	−0.06	(− 0.14 to 0.02)	0.13

*AUDIT* Alcohol Use Disorders Identification Test, *CES-D* Center for Epidemiological Studies Depression, *CI* confidence interval, *GEMS* Gender-Equitable Men Scale, *SD* standard deviation, *SRPS* Sexual Relationship Power Scale

A sub-analysis, using latent class analysis, explored whether the Sonke CHANGE intervention was more or less effective for subgroups of men.

Figure 4 shows the three distinct groups of men. Class 1 men displayed anti-social tendencies and used high levels of violence against partners and non-partners and engaged in risky sexual behaviours. Class 2 men used less violence against partners and non-partners but still engaged in high levels of risky sexual behaviours. Class 3 men used much less violence against partners and non-partners. The three-class model was chosen based on the measures of fit described in Supplementary material. Table 5 shows that Class 3 men living in intervention clusters, and potentially being exposed to the CHANGE intervention, had reduced their use of sexual violence against partners and had reduced use of severe IPV, although these differences did not reach statistical significance. However, men in Class 1 increased use of sexual IPV and severe IPV after the intervention had been implemented; again, these differences did not reach statistical significance.

**Table 5:** Differential treatment effects across latent subgroups

		Odds ratio	SE	95% CI		*P*(z)
Physical abuse	*Class 1*	1.03	0.43	0.46	2.32	0.94
	*Class 2*	1.78	0.83	0.71	4.44	0.22
	*Class 3*	0.86	0.33	0.40	1.82	0.68
Sexual abuse	*Class 1*	1.27	0.40	0.69	2.37	0.44
	*Class 2*	2.00	1.19	0.62	6.44	0.25
	*Class 3*	0.90	0.47	0.32	2.50	0.84
Severe IPV	*Class 1*	1.13	0.35	0.62	2.08	0.69
	*Class 2*	1.55	0.78	0.58	4.15	0.38
	*Class 3*	0.81	0.35	0.35	1.88	0.63

*CI* confidence interval, *IPV* interpersonal violence, *SE* standard error

## Discussion

We conducted a cluster randomised controlled trial to determine the effectiveness of the Sonke CHANGE intervention to prevent men’s use of sexual and or physical violence against an intimate partner and reduce the severe IPV perpetration by men living in a peri-urban South African settlement over 2 years of follow-up. We found that the intervention did not significantly affect any of the primary or secondary outcomes. There was no effect on men’s past year use of physical or sexual IPV or a reduction in severe IPV. There were also no differences in perpetration of non-partner rape perpetration, gender attitudes, use of transactional sex, parenting or social cohesion between intervention and control communities. Harmful use of alcohol may have worsened in the intervention communities over the 2 years of follow-up, although this was not statistically significant. The findings of the evaluation therefore did not support the core of the theory of change, that an intervention with multiple community mobilisation activities aimed at social norms change, could gain enough traction in a poor peri-urban community to catalyse changes in gender attitudes and social norms in order to reduced perpetration of IPV.

A sub-analysis using classes derived through LCA suggested that the CHANGE intervention may have had more impact reducing sexual and severe IPV among men who were least violent at baseline, as all the three primary outcomes’ had odds ratios that were < 1.00 for men in the least violent class. By contrast, odds ratios were all elevated for men in the most violent class, notwithstanding the overlapping confidence intervals. Thus, the intervention may have led to increased sexual violence among men who displayed anti-social and other aggressive and exploitative behaviour at baseline; alternatively, the intervention or research may have provoked these men to disclose more sexual violence. We cannot rule out the possibility of over-reporting by this subgroup of men, or under-reporting by less violent men; however, the very high levels of violence reported were similar to those found in a trial conducted in a similar settlement in eThekwini, South Africa [4].

Although many of the community outreach activities took place as planned, there were shortcomings, particularly in mobilising the community as was observed through the limited number of CATs active in any month (n = 18) and the very limited roll out of the advocacy campaign [34]. Our monitoring and evaluation data show that the target of reaching 60% of eligible men in each of the intervention clusters was achieved in some clusters but it is unclear whether the 40% target of reaching the same men more than once was met. Most community members were reached through door-to-door discussions which might be with one person or a small gathering of people and could be short, 20 min or less, or slightly longer, up to 1 h, and focused on sharing information about rights and gender equality with a view to recruiting participants for a workshop. A smaller proportion of men were reached through the more intensive 2-day workshops which used participatory learning approaches to explore and reflect upon gender-inequitable attitudes. There were a series of six workshops addressing different topics and few men attended multiple 2-day workshops, progressing through the full 72 h CHANGE workshop curriculum [34]. Many contextual factors contributed to the limited attendance of the full workshops series and also prevented men from attending for the full 2 days, such as seeking employment. In addition, the planned advocacy activities, which aimed to hold local government to account, were not fully realised.

A very recent evaluation of the Sonke community mobilisation intervention, another adapted version of ‘One Man Can’, conducted in a rural part of South Africa, reported a significant increase in men’s gender equitable attitudes, unlike in our study. They too found no significant differences in men’s use of IPV between the intervention and control communities [19]. When comparing how the intervention was delivered in the two settings, we noted that there were a few differences in the activities delivered. For example, digital stories and street soccer were not implemented in the peri-urban setting due to contextual factors such as limitations in recreational spaces and higher levels of crime. The number of community action teams that were recruited and active in the rural setting vastly exceeded the numbers in our study setting. The reasons for this could include greater social cohesion, less in and out migration of community members and involvement in different livelihood strategies.

We were not able to collect data from women in the communities. We therefore have no way of assessing if they perceived their safety changed and violence reduced due to the intervention. As noted earlier, in Uganda, community-based interventions that used activism and mobilisation to change gender attitudes and social norms resulted in reductions in women’s experiences of IPV [15, 35]. However, men’s reported attitudes and behaviours around perpetration of physical and sexual IPV have been much harder to change. We do not know if there was a tangible benefit for women due to the intervention that our research strategy has been unable to detect.

Overall, reports of men’s use of IPV and non-partner rape in the peri-urban setting were higher than levels reported in other studies. Wagman and colleagues reported levels of perpetration of sexual and physical IPV at 3%–9% at any time period compared to 15.8%–40.2% in our study [15]. The very high levels of violence perpetration found at the baseline speak to the context in which the intervention was delivered. The peri-urban setting is characterised by poorer infrastructure, where many people live in informal housing, and there are limitations in service delivery. There is substantial in and out migration which negatively affects social cohesion and there are high levels of crime and all forms of violence. A different kind of intervention may be warranted in this setting, such as the Stepping Stones Creating Futures intervention, which was conducted in the per-urban setting in eThekwini. The intervention, conducted in small groups over a period of time, focused on livelihoods as well as participatory learning approaches, including critical reflection, role play and drama to build more gender-equitable relationships with better communication between partners [36, 37].

The sub-analysis using LCA suggested that the intervention may have had an effect in the hypothesised direction among men who used less IPV and non-partner sexual violence. Less violent men may be more open to messages that related to gender equality and rights as they may experience greater levels of ambivalence about their behaviours. Greater ambivalence in gender role ideology can mediate men’s use of IPV [38]. By contrast, since the intervention seeks to change men’s attitudes and beliefs about gender equality in order to bring about a change in gendered social norms, it is therefore unsurprising that the most violent, anti-social and hypermasculine men who have entrenched gender role ideologies, enacted through sexual behaviours such as having multiple partners as well as controlling their sexual partners, are less susceptible to change through this kind of intervention. Literature suggests that men who adhere more rigidly to traditional masculinity that endorses dominance are more likely to have gender inequitable attitudes and beliefs and more likely to use IPV and believe that it is justified [38–41].

The results of the CRT needs to be interpreted in the light of several limitations. The number of clusters was limited to 18 due to the geographic size and the nature of the peri-urban setting where the CRT was conducted. Contamination where participants living in control clusters attended some of the activities in intervention clusters could have occurred. Efforts were made to avoid this from occurring, but it was difficult to prevent entirely. The proportion of participants followed up is acceptable especially given the nature of the trial and the context, yet it is possible that the loss to follow-up may have affected the results, especially as those who could not be traced reported higher levels of sexual and sever IPV at baseline. We have very little information about men who were lost to follow-up and they could have left the community for positive reasons having benefitted from the intervention. It is possible that there were measurement errors in the primary study outcomes: baseline reporting of IPV in the past 12 months may have been overreported if some men gave lifetime and not the past 12 month reports of their use of IPV, which may have been corrected at endpoint; alternatively baseline reports of past 12 month perpetration of IPV may have been accurate and men may have later concealed some perpetration after reflection that it was socially undesirable. These findings are limited to the largely informal peri-urban setting where the study was conducted.

The intensity of implementation in each intervention cluster varied. There were differences in the number of workshops that were held. In addition, the number of CATs recruited were not evenly distributed across all intervention clusters. These shortcomings could have affected the extent to which communities were mobilised within each cluster. Further analysis to investigate the effectiveness of the intervention that draws on the levels of exposure to the intervention should be carried out.

For the sub-analysis using LCA, the analysis was underpowered as the study was not powered to detect significant differences between subgroups of men. Two years of follow-up and 18 months of intervention delivery may not have been long enough to see effect in this peri-urban community. Women were not interviewed and followed up in this study, which means that we have no idea whether the intervention benefitted the considerable numbers of women who participated in the activities.

## Conclusion

The Sonke CHANGE intervention, when implemented in a peri-urban settlement in Johannesburg, had a limited effect in reducing IPV perpetrated by male residents. Further analysis showed the Sonke CHANGE intervention may have had more impact on less violent men, but exposure to the intervention may have resulted in resistance among most violent men. Poverty, crime and high levels of exposure to violence by residents suggested important modifications would be needed for successful IPV prevention in this context. Future research could consider the impact of therapeutic interventions or sustained, more intensive efforts with highly violent men in settings that are characterised by high rates of poverty and mental health challenges.

## Supplementary information

**Additional file 1.Supplementary Table 1.** Meansures of fit for models one through three latent classes.

## Data Availability

Data will be made available by DFiD (UKAID) by the end of 2019.

## References

[1] Devries KM, Mak JY, García-Moreno C, Petzold M, Child JC, Falder G (2013). The global prevalence of intimate partner violence against women. Science.

[2] Jewkes R, Morrell R (2018). Hegemonic masculinity, violence, and gender equality: Using latent class analysis to investigate the origins and correlates of differences between men. Men Masculinities.

[3] Fulu E, Warner X, Miedema S, Jewkes R, Roselli T, Lang J. Why do some men use violence against women and how can we prevent it. Quantitative Findings from the United Nations Multi-Country Study on Men and Violence in Asia and the Pacific. Bankok: United Nations Development Programme, United Nations Population Fund, United Nations Women and United nations Volunteers; 2013.

[4] Gibbs A, Jewkes R, Willan S, Washington L (2018). Associations between poverty, mental health and substance use, gender power, and intimate partner violence amongst young (18-30) women and men in urban informal settlements in South Africa: A cross-sectional study and structural equation model. PLoS One.

[5] Ranganathan M, Knight L, Abramsky T, Muvhango L, Polzer Ngwato T, Mbobelatsi M, Ferrari G, Watts C, Stöckl H. Associations between women’s economic and social empowerment and intimate partner violence: Findings from a microfinance plus program in rural North West Province, South Africa. J Interpersonal Violence. 2019:0886260519836952. 10.1177/0886260519836952 PMC8276343 30913954

[6] Groes-Green C (2009). Hegemonic and subordinated masculinities: Class, violence and sexual performance among young Mozambican men. Nord J Afr Stud.

[7] Hatcher AM, Stöckl H, McBride R-S, Khumalo M, Christofides N (2019). Pathways from food insecurity to intimate partner violence perpetration among peri-urban men in South Africa. Am J Prev Med.

[8] Hatcher AM, Gibbs A, Jewkes R, McBride R-S, Peacock D, Christofides N (2019). Effect of childhood poverty and trauma on adult depressive symptoms among young men in peri-urban South African settlements. J Adolesc Health.

[9] Bourgois P (1996). In search of masculinity: violence, respect and sexuality among Puerto Rican crack dealers in East Harlem. Br J Criminol.

[10] Wood K, Lambert H, Jewkes R (2007). "Showing roughness in a beautiful way": talk about love, coercion, and rape in South African youth sexual culture. Med Anthropol Q.

[11] World Health Organization. Engaging men and boys in changing gender-based inequity in health: Evidence from programme interventions. Geneva: 2007.

[12] Verma RK, Pulerwitz J, Mahendra VS, Khandekar S, Singh AK, Das SS, Mehra S, Nura A, Barker G. Promoting gender equity as a strategy to reduce HIV risk and gender-based violence among young men in India. Horizons Final Report. Washington, DC: Population Council; 2008.

[13] Peacock D (2013). South Africa's Sonke Gender Justice Network: Educating men for gender equality. Agenda.

[14] Abramsky T, Devries KM, Michau L, Nakuti J, Musuya T, Kyegombe N, Watts C (2016). The impact of SASA!, a community mobilisation intervention, on women's experiences of intimate partner violence: secondary findings from a cluster randomised trial in Kampala, Uganda. J Epidemiol Commun Health.

[15] Wagman JA, Gray RH, Campbell JC, Thoma M, Ndyanabo A, Ssekasanvu J, Nalugoda F, Kagaayi J, Nakigozi G, Serwadda D (2015). Effectiveness of an integrated intimate partner violence and HIV prevention intervention in Rakai, Uganda: analysis of an intervention in an existing cluster randomised cohort. Lancet Glob Health.

[16] Dworkin SL, Colvin C, Hatcher A, Peacock D (2012). Men’s perceptions of women’s rights and changing gender relations in South Africa: Lessons for working with men and boys in HIV and antiviolence programs. Gend Soc.

[17] Van den Berg W, Hendricks L, Hatcher A, Peacock D, Godana P, Dworkin S (2013). ‘One Man Can’: shifts in fatherhood beliefs and parenting practices following a gender-transformative programme in Eastern Cape, South Africa. Gender Dev.

[18] Lippman SA, Maman S, MacPhail C, Twine R, Peacock D, Kahn K, Pettifor A (2013). Conceptualizing community mobilization for HIV prevention: implications for HIV prevention programming in the African context. PLoS One.

[19] Pettifor A, Lippman SA, Gottert A, Suchindran CM, Selin A, Peacock D, Maman S, Rebombo D, Twine R, Gómez-Olivé FX (2018). Community mobilization to modify harmful gender norms and reduce HIV risk: results from a community cluster randomized trial in South Africa. J Int AIDS Soc.

[20] Christofides NJ, Hatcher AM, Pino A, Rebombo D, McBride RS, Anderson A, Peacock D (2018). A cluster randomised controlled trial to determine the effect of community mobilisation and advocacy on men’s use of violence in periurban South Africa: study protocol. BMJ Open.

[21] Hatcher AM, McBride R-S, Rebombo D, Munshi S, Khumalo M, Christofides N. Process evaluation of a community mobilization intervention for preventing men’s partner violence use in peri-urban South Africa. Eval Program Plan. 2020;78:101727. 10.1016/j.evalprogplan.2019.101727 PMC7264430 31639542

[22] Fulu E, Jewkes R (2014). Replicating the UN Multi-Country Study on men and violence: Understanding why some men use violence against women and how we can prevent it..

[23] Fulu E, Warner X, Miedema S, Jewkes R, Roselli T, Lang J (2013). Why do some men use violence against women and how can we prevent it? Findings from the UN Multi-country study on men and violene in Asia and the Pacific..

[24] Jewkes R, Sikweyiya Y, Morrell R, Dunkle K (2011). The relationship between intimate partner violence, rape and HIV amongst South African men: a cross-sectional study. PLoS One.

[25] Saunders JB, Aasland OG, Babor TF, de la Fuente JR, Grant M (1993). Development of the Alcohol Use Disorders Identification Test (AUDIT): WHO Collaborative Project on Early Detection of Persons with Harmful Alcohol Consumption--II. Addiction.

[26] Pulerwitz J, Barker G (2008). Measuring attitudes towards gender norms among young men in Brazil: Development and psychometric evaluation of the GEM scale. Men Masculinities.

[27] MRC (2010). War at Home: Preliminary findings of the Gauteng Gender Violence Prevalence Study.

[28] Pulerwitz J, Amaro H, De Jong W, Gortmaker SL, Rudd R (2002). Relationship power, condom use and HIV risk among women in the USA. AIDS Care.

[29] Straus MA, Hamby SL, Finkelhor D, Moore DW, Runyan D (1998). Identification of child maltreatment with the Parent-Child Conflict Tactics Scales: Development and psychometric data for a national sample of American parents. Child Abuse Negl.

[30] Jewkes R, Nduna M, Levin J, Jama N, Dunkle K, Puren A, Duvvury N (2008). Impact of stepping stones on incidence of HIV and HSV-2 and sexual behaviour in rural South Africa: cluster randomised controlled trial. BMJ.

[31] Radloff LS (1977). The CES-D scale a self-report depression scale for research in the general population. Appl Psychol Meas.

[32] Deitchler M, Ballard T, Swindale A, Coates J (2010). Validation of a measure of household hunger for cross-cultural use.

[33] Hayes RJ, Moulton LH (2009). Cluster Randomised Trials.

[34] Hatcher AM, McBride R-S, Rebombo D, Munshi S, Khumalo M, Christofides N (2020). Process evaluation of a community mobilization intervention for preventing men’s partner violence use in peri-urban South Africa. Eval Program Plann.

[35] Abramsky T, Devries K, Kiss L, Nakuti J, Kyegombe N, Starmann E, Cundill B, Francisco L, Kaye D, Musuya T (2014). Findings from the SASA! Study: a cluster randomized controlled trial to assess the impact of a community mobilization intervention to prevent violence against women and reduce HIV risk in Kampala, Uganda. BMC Med.

[36] Jewkes R, Gibbs A, Jama-Shai N, Willan S, Misselhorn A, Mushinga M, Washington L, Mbatha N, Skiweyiya Y (2014). Stepping Stones and Creating Futures intervention: shortened interrupted time series evaluation of a behavioural and structural health promotion and violence prevention intervention for young people in informal settlements in Durban, South Africa. BMC Public Health.

[37] Gibbs A, Washington L, Willan S, Ntini N, Khumalo T, Mbatha N, Sikweyiya Y, Shai N, Chirwa E, Strauss M (2017). The Stepping Stones and Creating Futures intervention to prevent intimate partner violence and HIV-risk behaviours in Durban, South Africa: study protocol for a cluster randomized control trial, and baseline characteristics. BMC Public Health.

[38] Santana MC, Raj A, Decker MR, La Marche A, Silverman JG (2006). Masculine gender roles associated with increased sexual risk and intimate partner violence perpetration among young adult men. J Urban Health.

[39] Gage AN, Lease SH. An exploration of the link between masculinity and endorsement of IPV myths in American men. J Interpersonal Violence. 2018:0886260518818430. 10.1177/0886260518818430 30547718

[40] Reed E, Silverman JG, Welles SL, Santana MC, Missmer SA, Raj A (2009). Associations between perceptions and involvement in neighborhood violence and intimate partner violence perpetration among urban, African American men. J Community Health.

[41] Fleming PJ, McCleary-Sills J, Morton M, Levtov R, Heilman B, Barker G (2015). Risk factors for men's lifetime perpetration of physical violence against intimate partners: results from the international men and gender equality survey (IMAGES) in eight countries. PLoS One.

