# Correction to: Effectiveness of a multi-level intervention to reduce men’s perpetration of intimate partner violence: a cluster randomised controlled trial

**DOI:** 10.1186/s13063-020-04386-z

**Published:** 2020-06-16

**Authors:** Nicola J. Christofides, Abigail M. Hatcher, Dumisani Rebombo, Ruari-Santiago McBride, Shehnaz Munshi, Angelica Pino, Nada Abdelatif, Dean Peacock, Jonathan Levin, Rachel K. Jewkes

**Affiliations:** 1 Faculty of Health Sciences, School of Public Health, University of the Witwatersrand, 27 St Andrews Rd, Parktown, Johannesburg, 2193 South Africa; 2 Division of HIV, Infectious Disease, and Global Medicine, University of California, San Francisco, USA; 3 Sonke Gender Justice, Juta Street, Braamfontein, Johannesburg, South Africa; 4 South African Medical Research Council, 1 Soutpansberg Road, Pretoria, South Africa

**Correction to: Trials (2020) 21:359**

**https://doi.org/10.1186/s13063-020-4185-7**

Following publication of the original article [1], the authors identified an error in Fig. 2. The correct Fig. 2 is given below.

**Fig. 2:**
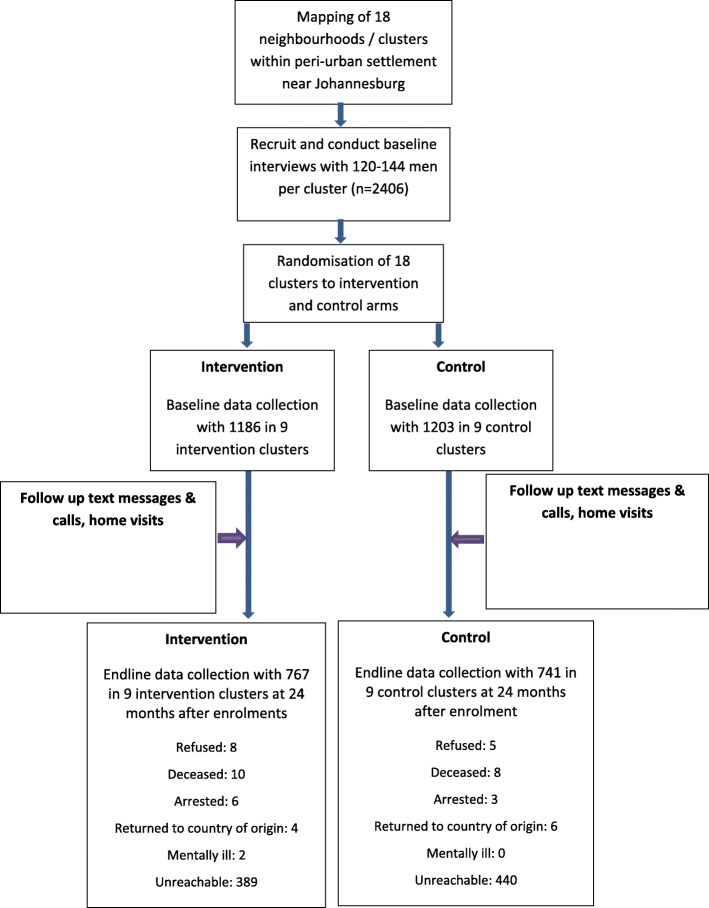
Flow diagram showing baseline recruitment, allocation (2016) and endline retention (2018)

The original article has been corrected.

## References

[1] Christofides NJ (2020). Effectiveness of a multi-level intervention to reduce men’s perpetration of intimate partner violence: a cluster randomised controlled trial. Trials.

