# Supplementary material for: Effectiveness of a multi-level intervention to reduce men’s perpetration of intimate partner violence: a cluster randomised controlled trial
Source: Trials. 2020 Apr 25;21:359. doi: 10.1186/s13063-020-4185-7 (PMC7183134; doi:10.1186/s13063-020-4185-7)
Supplement: Supplementary file 1 — Additional file 1.Supplementary Table 1. Meansures of fit for models one through three latent classes. [file 13063_2020_4185_MOESM1_ESM.docx]

Supplementary material

L^2^ shows that 2 and 3 latent class models have adequate fits to the data. The aBIC and CAIC are smaller for 3 latent classes (aBIC = 22 927, CAIC = 22 919) than for 2 latent classes, but entropy was higher for 2 latent classes (0.79). The LMR LRT compares the improvement in fit between neighbouring nested class models, where the n-class is compared to n-1-class and determines whether there is an improvement in fit for including one more class. The LMR LRT has a p-value <0.001, which suggests that the 3 latent class model is an improvement to the 2-class model. More latent classes were explored (not shown here), however, a few variables had significant bivariate residuals meaning that the local independence assumption was invalid. This assumption was relaxed by modelling the covariances between the variables and this resulted in only up to 3 classes being useful.

Supplementary table 1: Measures of fit for models with one through x latent classes

| **Number of classes (n)** | **LRT p-value (a)** | **aBIC (b)** | **CAIC** | **Entropy** | **Lo-Mendell-Rubin adjusted LRT p-value (c)** |
| --- | --- | --- | --- | --- | --- |
| 1 | 0.0000 | 25,837 | 25,834 |  |  |
| 2 | 0.0868 | 23,109 | 23,104 | 0.79 | 0.0000 |
| 3 | 0.9979 | 22,927 | 22,919 | 0.70 | 1 |
|  |  |  |  |  |  |
|  | *(a) Significant p-value indicates lack of model fit in absolute terms* | | | | |
|  | *(b) Lower value suggests more optimal balance between model fit and parsimony* | | | | |
|  | *(c) Non-significant p-value indicates rejecting the null hypothesis of n-1-class model for alternative n-class model* | | | | |
